# Public health round-up

**DOI:** 10.2471/BLT.22.010222

**Published:** 2022-02-01

**Missing targets on neonatal and infant mortality**

A family physician trains community health workers in maternal health care as part of a UNICEF-supported initiative in Yemen. According to the latest estimates released by the United Nations Inter-agency Group for Child Mortality Estimation, more than 50 countries risk missing the sustainable development goal target for under-five mortality of 25 or fewer deaths per 1000 live births by 2030, while more than 60 countries risk missing the neonatal mortality target of 12 or fewer deaths per 1000 live births by the same deadline.

**Figure:**
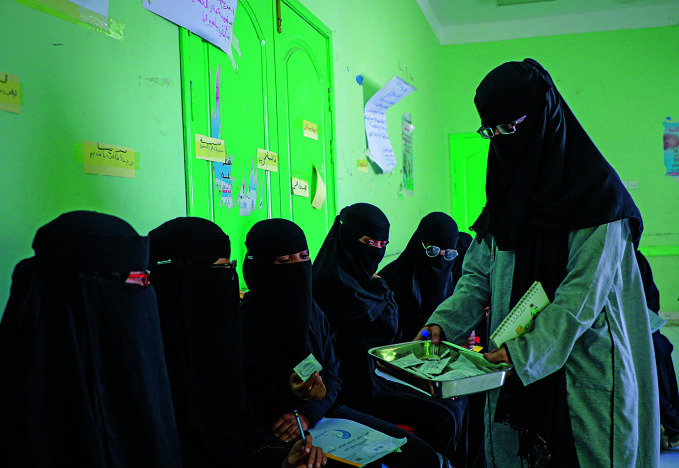
UNICEF/Moohialdin Fuad

## Tonga volcano

The Hunga Tonga Hunga Ha’apai volcano in Tonga erupted on 15 January, generating a tsunami that hit coastal areas. The loss of communications limited initial reports, but the waterfront of the Tongan capital, Nuku’alofa, appeared to have been seriously damaged with rocks and debris pushed inland by the tsunami. There was also significant infrastructural damage around Tongatapu, Tonga’s main island, and a 1–2 cm blanket of volcanic ash covered the majority of the country, impacting water and food supplies.

The Tongan government’s response included deploying a naval vessel to the Ha’apai islands carrying World Health Organization (WHO)-trained Tonga Emergency Medical Assistance Team to help treat any people who may have been injured.

On 17 January, the Pacific Humanitarian Team, comprising United Nations (UN) agencies, the Red Cross movement and Oxfam began coordinating a response that included the provision of in-country support. 

According to WHO’s Country Liaison Officer for Tonga, Dr Yutaro Setoya, around 100 houses had been damaged and 50 completely destroyed on the main island of Tongatapu. Two deaths had been reported and many people remained displaced. All health facilities on Tongatapu were reported to be fully functioning and clean-up efforts had been initiated.

https://bit.ly/3Kwv6ee

## New COVID-19 vaccines needed

The composition of current coronavirus disease 2019 (COVID-19) vaccines needs to be updated to ensure that they continue to provide WHO-recommended levels of protection (at least 70% on population basis) against infection and disease associated with variants of concern (VOCs), including Omicron and future VOCs.

This is according to the WHO-convened Technical Advisory Group on COVID-19 Vaccine Composition, an independent group of experts tasked with reviewing evidence and assessing the implications of emerging VOCs on the performance of COVID-19 vaccines.

In an 11 January statement, the group recommended the development of COVID-19 vaccines based on strains that are genetically and antigenically close to circulating VOCs, protect against severe disease and death, reduce infectivity and elicit lasting immune responses to reduce the need for successive booster doses. The group also urged broader access globally to current COVID-19 vaccines for primary series and booster doses which may mitigate the emergence and impact of new VOCs.

https://bit.ly/3qn8bd5

## Inequitable COVID-19 vaccine access

The Independent Allocation of Vaccines Group (IAVG) called for the achievement of 70% coverage with COVID-19 vaccines in all countries by mid-2022 as part of a set of recommendations to make the allocation of COVID-19 vaccines more equitable and effective.

The IAVG is tasked with validating the allocation of COVID-19 vaccine through COVAX, the vaccines pillar of the Access to COVID-19 Tools (ACT) Accelerator, a global collaboration designed to ensure equitable access to COVID-19 tests, treatments and vaccines.

In a 23 December statement, the IAVG expressed concern that the use of available vaccines is not aligned with the Strategy to Achieve Global COVID-19 Vaccination by Mid-2022 and called on all countries to work with COVAX to optimize the use of the growing vaccine supply. The IAVG also encouraged high-coverage countries to establish complementary, “dual-track” approaches that take account of domestic and international coverage goals.

In related news, WHO called on all citizens to demand that governments and pharmaceutical companies share health tools globally and bring an end to the death and disease caused by the pandemic, limit new variants and drive a global economic recovery. The 16 January appeal noted that despite COVAX delivering its billionth vaccine, more work was needed to ensure equitable access. As of 13 January, 36 of the 194 WHO Member States had vaccinated fewer than one in 10 of their populations, and 88 fewer than four in 10.

https://bit.ly/3FW6A3u

https://bit.ly/3tAw6YD

## WHO lists new COVID-19 vaccine

WHO issued an emergency use listing (EUL) for the Covovax vaccine (NVX-CoV2373), expanding the basket of WHO-validated vaccines against the severe acute respiratory syndrome coronavirus 2 (SARS-CoV-2) virus.

Covovax is derived from an engineered baculovirus containing a gene for a modified SARS-CoV-2 spike protein and, stable at 2 to 8 °C, is suitable for storage in normal commercial refrigerators. The vaccine is produced by the Serum Institute of India under license from American biotechnology company Novavax and is included in the COVAX portfolio.

An EUL listing assures the quality, safety and efficacy of the vaccine and is a prerequisite for COVAX vaccine supply. It also allows countries to expedite their own regulatory approval to import and administer the vaccine.

https://bit.ly/3JBxi3s

## WHO recommends two new COVID-19 treatments

WHO strongly recommended the use of rheumatoid arthritis drug baricitinib in combination with corticosteroids for the treatment of patients with severe or critical COVID-19. Announced by WHO’s Guideline Development Group on 13 January, the recommendation is based on evidence that the drug improves survival and reduces the need for ventilation, with no observed increase in adverse effects.

The group also made a conditional recommendation for the use of the monoclonal antibody sotrovimab in patients with non-severe COVID-19, but only in those at highest risk of hospitalization. The recommendations are based on new evidence from seven trials involving over 4000 patients with non-severe, severe and critical COVID-19 infection.

https://bit.ly/33N7xwS

## Democratic Republic of the Congo meningitis outbreak ends

The government of the Democratic Republic of the Congo reported that the meningitis outbreak declared on 7 September 2021 was over. The outbreak began in Tshopo Province, probably starting in early June 2021. A total of 2662 people were reported to have been infected, 205 of whom died.

According to a 24 December report, WHO supported the national and provincial health authorities in its outbreak response which involved setting up local health emergency management teams, bolstering disease surveillance, implementing vaccination drives and providing medical care.

“Ending this outbreak under difficult circumstances and amid the COVID-19 pandemic is no mean feat by the national authorities, but we must invest more to better detect, prevent and lessen the debilitating impact of this disease,” said Dr Matshidiso Moeti, WHO Regional Director for Africa.

https://bit.ly/3HyY1vK

**Cover photo**

Their protective masks in place, 5th grade students from Kendriya Vidyalaya school in east Delhi, India, take the National Achievement Survey 2021.

**Figure:**
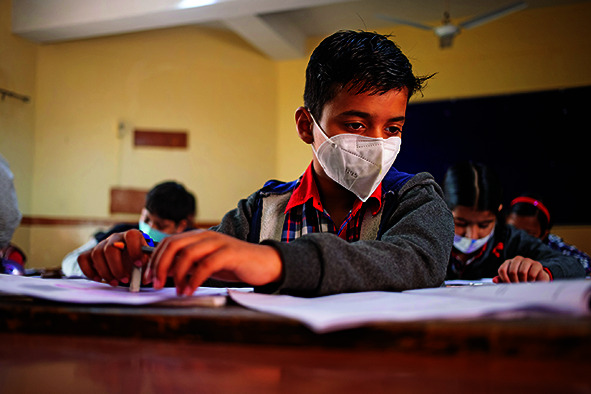
UNICEF/Zishaan Akbar Latif

## Missing newborn/child mortality targets

The world remains significantly off track to meet the sustainable development goal targets on ending the preventable deaths of newborns and children under five, according to the latest estimates released by the UN Inter-agency Group for Child Mortality Estimation.

According to a 20 December report, without immediate action, more than 50 countries risk missing the under-five mortality target (25 or fewer deaths per 1000 live births by 2030) and more than 60 countries will miss the neonatal mortality target (12 or fewer deaths per 1000 live births by 2030).

More than 5 million children died before their fifth birthday in 2020 alone, along with 2.2 million children and young people aged 5 to 24. The report also highlights the urgent need to invest in strengthening data systems to track newborn and child health and mortality in low- and middle-income countries, two thirds of which have had no reliable mortality data in the past three years.

https://bit.ly/3r8pJc0

## Mental health support for refugees

A WHO-developed self-help psychological intervention known as Self-Help Plus (SH+) was associated with a decline in the onset of mental disorders among Syrian refugees in Turkey, according to a study published in World Psychiatry on 11 January.

SH+ is based on a form of cognitive behavioural therapy and consists of a pre-recorded audio course and complementary book, providing information on managing stress and guiding participants through individual exercises and small group discussions.

Half of the 624 adult participants received SH+ and routinely delivered social support and/or care, while half received social support and/or care alone. The study found that SH+ participants were significantly less likely to have any mental disorders at six-month follow-up compared to the control group (22% versus 41%).

https://bit.ly/3fkcPCp

**Looking ahead**

4 February. World Cancer Day: close the care gap. https://bit.ly/3HAjkgt

15–17 February. Global Disability Summit. https://bit.ly/3plrwd1

17–18 March. High-level meeting on health and migration in the WHO European Region. https://bit.ly/3Des3T6

